# Late‐Onset Bone Metastasis 46 Years After Initial Surgery for Pheochromocytoma: A Case Report

**DOI:** 10.1002/iju5.70086

**Published:** 2025-08-13

**Authors:** Fumio Ishizaki, Kaede Hiruma, Yusuke Tani, Hideaki Sugino, Tatsuro Sanami, Tsutomu Anraku, Masahiro Ikeda, Masayuki Tasaki, Kazuhide Saito, Yoshihiko Tomita

**Affiliations:** ^1^ Department of Urology, Molecular Oncology, Graduate School of Medical and Dental Sciences Niigata University Niigata Japan; ^2^ Division of Molecular and Diagnostic Pathology, Niigata University Graduate School of Medical and Dental Sciences Niigata University Niigata Japan; ^3^ Department of Diagnostic Pathology Saiseikai Niigata Kenoh Kikan Hospital Niigata Japan

**Keywords:** bone metastasis, late recurrence, pheochromocytoma

## Abstract

**Introduction:**

Pheochromocytoma is a rare catecholamine‐producing tumor with metastatic potential. Recurrence after more than 40 years is exceptionally rare.

**Case Presentation:**

During evaluation for ischemic colitis, a 71‐year‐old woman was found to have multiple bone metastases, possibly linked to catecholamine excess. She had undergone left adrenalectomy for pheochromocytoma at age 25. Bone biopsy confirmed metastatic pheochromocytoma, and immunohistochemical findings were similar to the original tumor. Urinary metanephrine and normetanephrine were markedly elevated. She declined systemic therapy and has remained clinically stable for 6 years, with her blood pressure well controlled on doxazosin.

**Conclusion:**

This case illustrates a recurrence 46 years after adrenalectomy, potentially representing the longest reported interval to date. It highlights the silent and indolent nature of some metastatic pheochromocytomas and underscores the necessity of lifelong follow‐up. The patient's stable course also emphasizes the clinical heterogeneity of metastatic pheochromocytoma and supports the need for individualized follow‐up and treatment strategies.

**Summary**

- We report a rare case of bone metastasis occurring 46 years after adrenalectomy for pheochromocytoma, representing the longest disease‐free interval ever documented.
- This case highlights the potential for ultra‐late recurrence and underscores the importance of considering long‐term follow‐up in patients with pheochromocytoma.

**Abbreviations**
CT: computed tomography
MIBG: metaiodobenzylguanidine
MRI: magnetic resonance imaging

## 1 Introduction

Pheochromocytomas and paragangliomas (PPGL) are rare neuroendocrine tumors derived from chromaffin cells. Although previously considered benign unless metastatic, the current WHO classification defines all PPGLs as having metastatic potential [1]. The reported risk of metastasis is 10%–20% for pheochromocytomas and up to 50% for paragangliomas [2].

Common metastatic sites include the bone, liver, lungs, and lymph nodes. Most recurrences are observed within 10 years; but some cases recur after much longer intervals [3, 4]. Thus, international guidelines recommend at least 10 years of follow‐up for all patients with PPGL and lifelong follow‐up for those at high risk, such as younger patients, those with genetic syndromes, large tumors, or paragangliomas [5].

Herein, we present a case of metastatic pheochromocytoma detected 46 years after initial adrenalectomy.

## 2 Case

**FIGURE 1:**
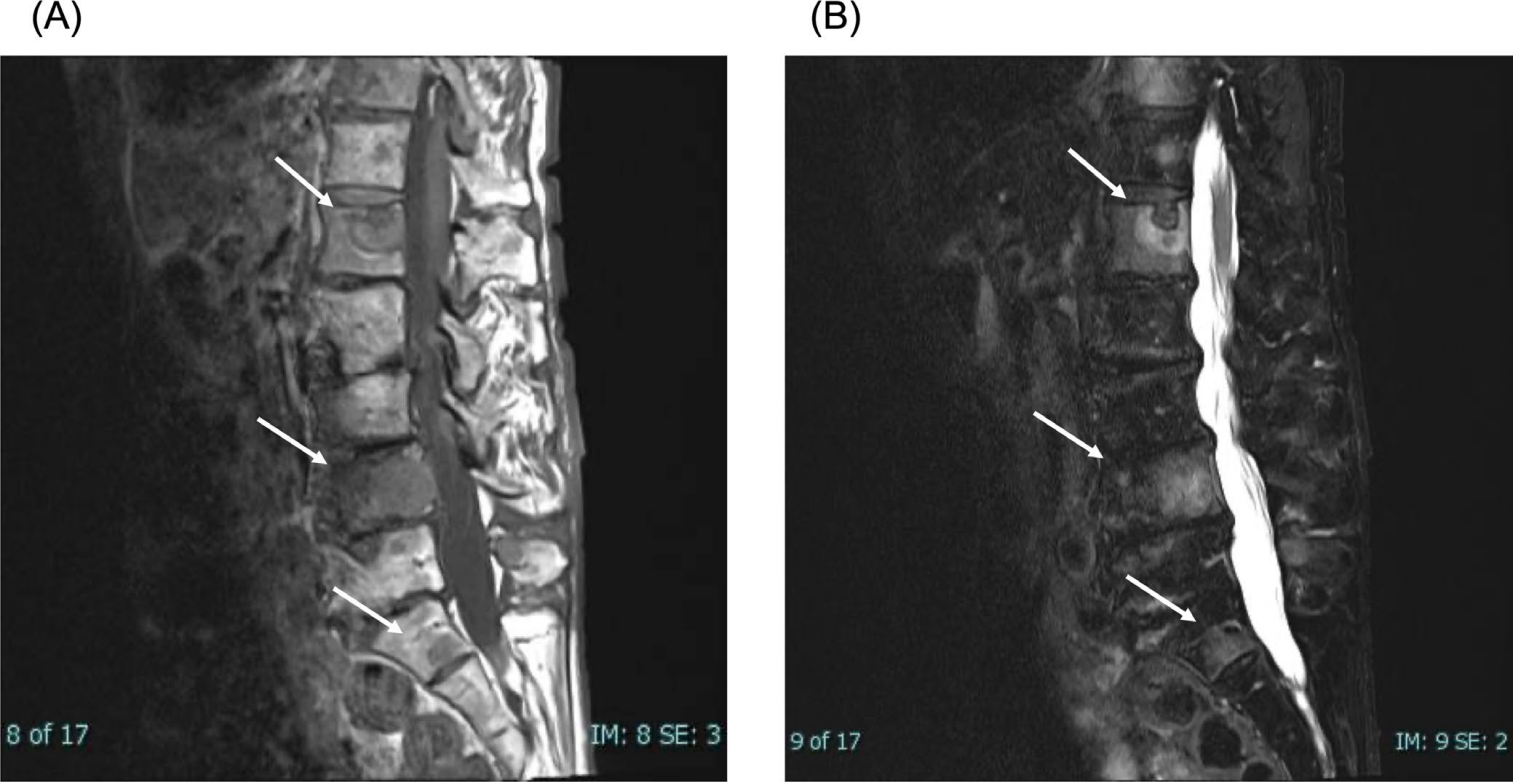
MRI findings of spinal metastases. (A) Sagittal T1‐weighted MRI showing hypointense lesions in the L1, L4 vertebrae and the sacrum. (B) Sagittal T2‐weighted MRI demonstrating corresponding hyperintense lesions in the same regions, consistent with bone metastases.

A 71‐year‐old woman presented with ischemic colitis, which may have been a symptom of catecholamine excess. She had undergone left adrenalectomy at age 25 following a diagnosis of pheochromocytoma. After several years of follow‐up, she was lost to long‐term monitoring. During evaluation, CT examination incidentally revealed osteolytic lesions. MRI showed bone metastases in Th11, L1, and L4 vertebrae; sacrum; and left iliac bone (Figure 1). The colitis resolved with conservative management.

**FIGURE 2:**
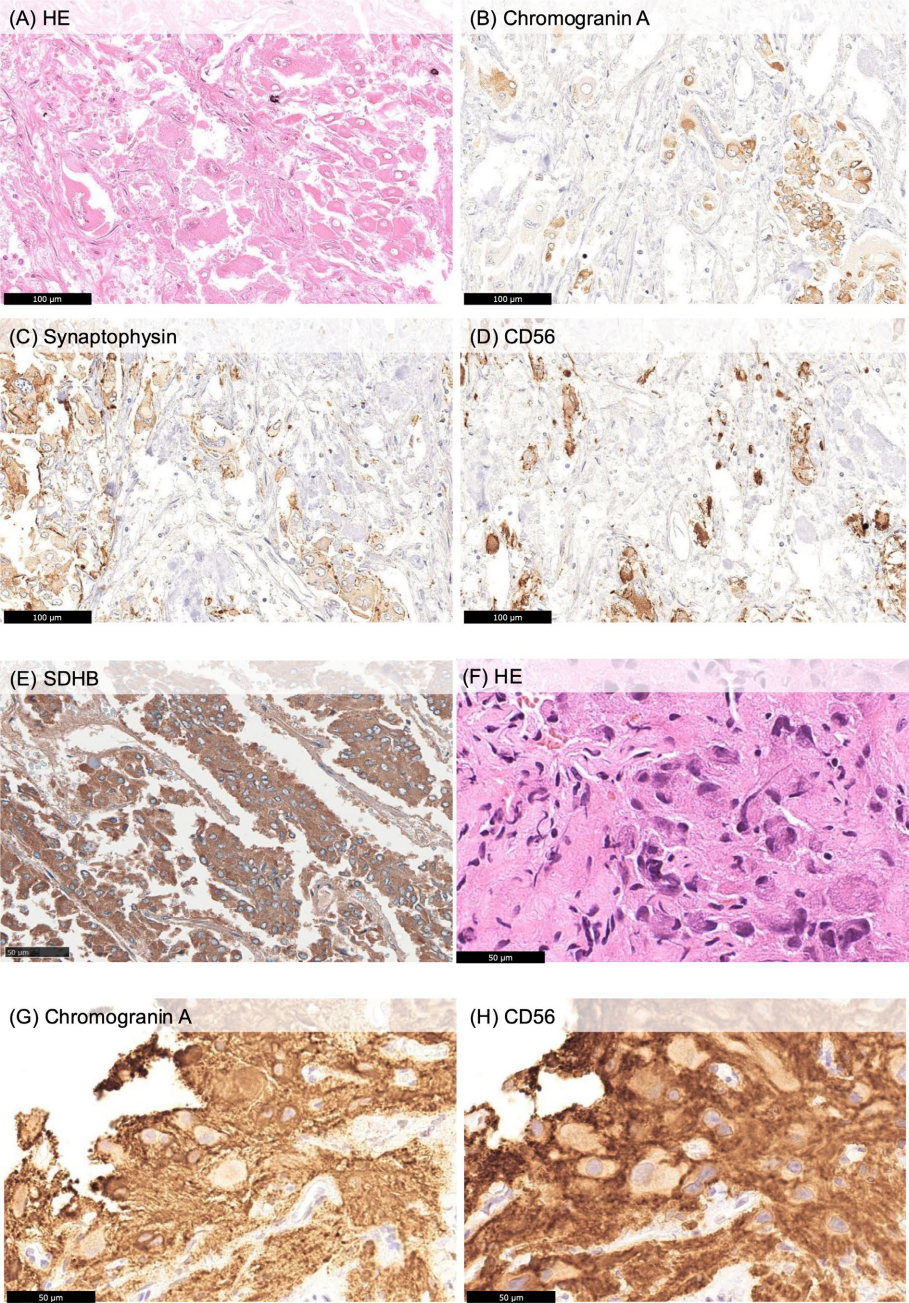
Histological and immunohistochemical findings. (A–E) Primary adrenal pheochromocytoma resected at age 25: Hematoxylin and eosin (HE) staining (A), and immunohistochemical staining for chromogranin A (B), synaptophysin (C), CD56 (D) and SDHB (E). (F–H) Bone metastasis biopsy specimen: HE staining (F) and immunohistochemical staining for chromogranin A (G) and CD56 (H).

Biopsy of a vertebral lesion was performed; during which the patient experienced significant blood pressure elevation. During biopsy, the systolic blood pressure rose up to 260 mmHg, which was controlled using a continuous infusion of calcium channel blockers. Histologically, the lesion exhibited a Zellballen pattern. The tumor cells tested positive for chromogranin A and CD56; however, excessive synaptophysin staining prevented accurate evaluation. The initial adrenal tumor, identified at age 25, was re‐examined and showed positivity for chromogranin A, synaptophysin, and CD56. The tumor also showed preserved SDHB expression, suggesting the absence of an SDHB mutation (Figure 2). The initial tumor demonstrated a PASS (Pheochromocytoma of the Adrenal gland Scaled Score) of 5 points and a GAPP (Grading of Adrenal Pheochromocytoma and Paraganglioma) score of 2 or 3 points, as the catecholamine secretion status was unclear [6, 7]. Unfortunately, due to the long interval, the medical records and images from the initial surgery were no longer available, and the tumor size at that time could not be determined.

**FIGURE 3:**
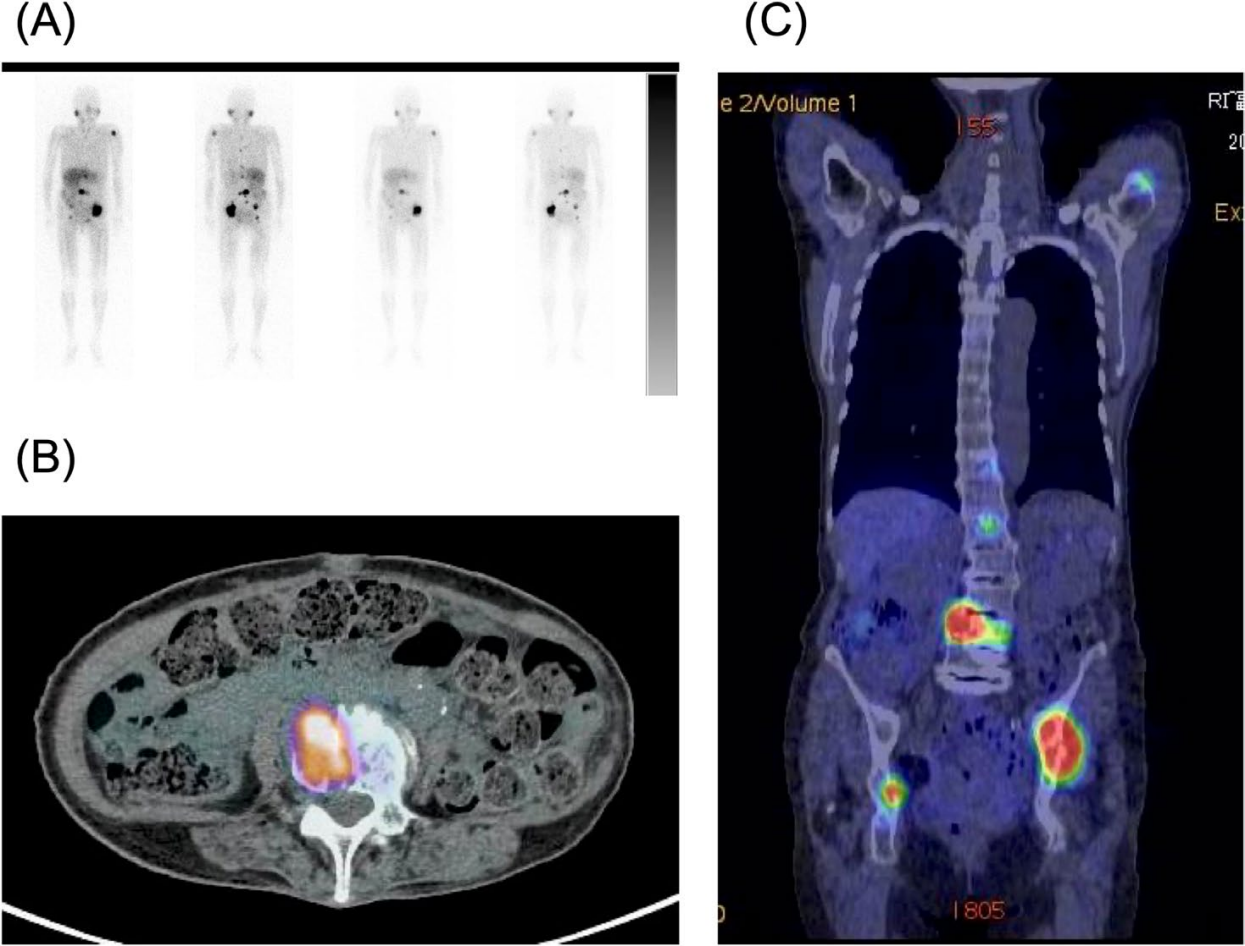
Imaging findings of metastatic pheochromocytoma. (A) ^123^I‐MIBG scintigraphy showing abnormal tracer uptake in the thoracic and lumbar spine, sacrum, left humerus, and bilateral iliac bones. (B) Axial PET‐CT at the L4 level demonstrating increased FDG uptake in the vertebral lesion. (C) Coronal PET‐CT showing FDG‐avid lesions in multiple vertebrae and pelvic bones, corresponding to the MIBG‐avid regions.

Urinary catecholamine levels were elevated: metanephrine 1.46 mg/day (normal, 0.04–0.1 mg/day), and normetanephrine 8.26 mg/day (normal, 0.1–0.28 mg/day). ^123^I‐MIBG scintigraphy showed tracer accumulation in Th11, L1, and L4 vertebrae; sacrum; left humerus; and both iliac bones. PET‐CT also demonstrated abnormal uptake in the same locations (Figure 3).

**FIGURE 4:**
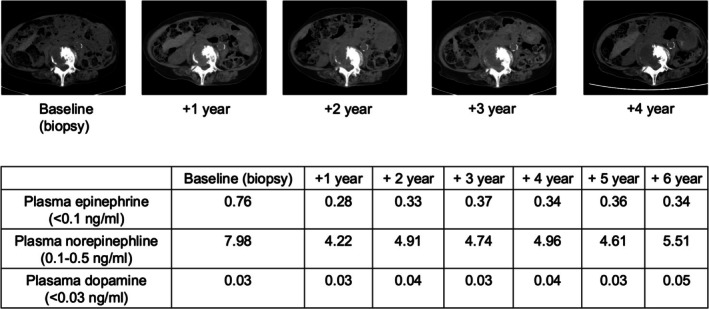
Follow‐up findings over 6 years after diagnosis of bone metastasis. Serial computed tomography (CT) images showing no significant progression of bone metastases from baseline (biopsy) to year 4. Plasma catecholamine levels remained stably elevated without further increase during the 6‐year observation period.

The patient chose to undergo observation without systemic therapy. Six years later, she remains stable, with her hypertension controlled on 2 mg/day of doxazosin. Genetic testing was not performed. During the 6‐year observation period after the diagnosis of bone metastasis, the patient underwent regular CT evaluations. These follow‐up CT scans showed no significant progression of the metastatic lesions. Additionally, plasma catecholamine levels remained stably elevated without further increase (Figure 4). These findings supported the continued decision for observation without systemic therapy.

## 3 Discussion

This case demonstrates one of the longest reported intervals between adrenalectomy and recurrence of pheochromocytoma. Although the metastatic lesions were found 46 years after surgery, the exact timing of recurrence is unclear. The extensive bone involvement and high catecholamine levels suggest the disease was active for several years before detection. This highlights the indolent nature of some PPGLs and reinforces the value of long‐term surveillance. If long‐term follow‐up had been maintained, it is likely that recurrence could have been detected before the onset of catecholamine‐related symptoms, such as ischemic colitis observed in this case.

The patient's stable condition over 6 years without treatment underscores the heterogeneity of metastatic PPGL. Some patients experience aggressive disease, whereas others have slow‐growing, indolent tumors [8]. This case also illustrates the marked heterogeneity in the clinical course of metastatic pheochromocytoma. Although some patients experience rapid progression shortly after surgery, others, like our patient, exhibit extremely delayed recurrence and indolent disease behavior even in the presence of multiple bone metastases. This underscores the importance of individualized therapeutic decision‐making, especially regarding the timing and necessity of systemic interventions. Although several treatment options were available at the time of recurrence—including cyclophosphamide, vincristine, and dacarbazine (CVD) chemotherapy, ^131^I‐MIBG radiotherapy, and metyrosine administration—the decision to proceed with observation was made after thorough multidisciplinary discussion. The patient's strong preference for non‐invasive management was respected, and given the indolent clinical behavior, observation without systemic therapy was considered an appropriate approach.

This variability highlights the importance of personalized follow‐up strategies. Although the initial adrenal tumor demonstrated a PASS score of 5 and a GAPP score of 2 or 3, recent updates in the classification of these tumors should be considered. According to the 2022 WHO Classification of Endocrine Tumors, all pheochromocytomas and paragangliomas are now regarded as having metastatic potential, and histologic scoring systems such as PASS and GAPP are no longer recommended as standalone predictors of malignancy [9]. Nevertheless, their use is not prohibited and may still offer supportive information when interpreted in conjunction with clinical findings, genetic background, and long‐term outcomes [3]. In our case, the late recurrence despite relatively low scores highlights the limitations of relying solely on these systems.

Notably, the patient was 25 years old at the time of the initial surgery, which alone places her in the high‐risk category that current guidelines recommend for lifelong surveillance, even though tumor size and genetic status could not be fully assessed. Given the high heritability of PPGL, genetic testing plays a critical role in the risk stratification and long‐term management of patients. Current international guidelines recommend considering genetic testing in all patients with PPGL [5, 10]. Although genetic testing was not performed in our case, it should be considered whenever possible, as it may influence follow‐up intensity, family counseling, and therapeutic planning. In our case, SDHB immunohistochemistry was positive, suggesting a lower likelihood of SDHB mutation, which is typically associated with more aggressive behavior.

In recent years, PPGLs have been stratified into three molecular clusters based on germline and somatic mutations: pseudohypoxia‐related (Cluster 1), kinase signaling‐related (Cluster 2), and Wnt signaling‐related (Cluster 3). Cluster 1 is further divided into subgroups associated with the TCA cycle and hypoxia pathways. Furthermore, recent advances in molecular classification, such as cluster‐based categorization of PPGL, have improved our understanding of their clinical behavior. Cluster 1 tumors (e.g., SDHB, VHL mutations) are associated with pseudohypoxia signaling and tend to exhibit aggressive features and higher metastatic potential, while Cluster 2 tumors (e.g., RET, NF1) follow kinase signaling pathways and often show a more indolent course [11, 12, 13]. These molecular classifications differ in tumorigenic mechanisms, catecholamine secretion patterns, optimal imaging approaches, and metastatic risk, and can assist in tailoring follow‐up strategies and therapeutic decisions [14, 15]. Moreover, accumulating evidence indicates that specific germline mutations may influence therapeutic response. For instance, SDHB‐mutated tumors have been reported to respond more favorably to cyclophosphamide, vincristine, and dacarbazine (CVD) chemotherapy, with significantly longer progression‐free survival compared to non‐SDHB tumors (23.7 vs. 5.2 months; *p* = 0.001) [16]. These findings underscore that genetic testing not only informs prognosis and surveillance but also facilitates individualized treatment planning, including therapeutic selection.

## 4 Conclusion

This case highlights the possibility of extremely late recurrence of pheochromocytoma. The patient experienced recurrence 46 years after the initial adrenalectomy, possibly representing the longest interval reported to date. This case highlights that recurrence can occur even after decades of apparent remission, often progressing silently and indolently. It is important to consider long‐term follow‐up in patients with pheochromocytoma.

This case demonstrates the diverse clinical behavior of metastatic pheochromocytoma and supports the need for personalized surveillance and treatment strategies.

## Ethics Statement

The study was approved by the Ethics Committee of Niigata University.

## Consent

Waiver of informed consent was granted by the Ethics Committee of Niigata University.

## Conflicts of Interest

The authors declare no conflicts of interest.
